# Evaluation of household coverage with long-lasting insecticidal nets in central Côte d’Ivoire

**DOI:** 10.1186/s12936-025-05335-4

**Published:** 2025-03-29

**Authors:** Colette Sih, Serge B. Assi, Benoit Talbot, Edouard Dangbenon, Manisha A. Kulkarni, Alphonsine A. Koffi, Ludovic P. Ahoua Alou, Louisa A. Messenger, Marius Gonse Zoh, Soromane Camara, Natacha Protopopoff, Raphael N’Guessan, Jackie Cook

**Affiliations:** 1 Faculty of Epidemiology and Population Health, Department of Infectious Disease Epidemiology, London School of Hygiene and Tropical Medicine, London, WC1E 7HT UK; 2 Institut Pierre Richet (IPR)/Institut National de Santé Publique (INSP), Bouaké, Côte d’Ivoire; 3 School of Epidemiology & Public Health, Faculty of Medicine, University of Ottawa, Ottawa, ON Canada; 4 Faculty of Infectious and Tropical Diseases, Disease Control Department, London School of Hygiene and Tropical Medicine, London, WC1E 7HT UK; 5 Department of Environmental and Occupational Health, School of Public Health, University of Nevada, Las Vegas, NV 89154 USA; 6 Health Interventions Unit, Department of Epidemiology and Public Health, Swiss Tropical & Public Health Institute, Kreuzstrasse 2, 4123 Allschwill, Switzerland; 7 Medical Research Council (MRC) International Statistics and Epidemiology Group, Department of Infectious Disease Epidemiology and International Health, London School of Hygiene and Tropical Medicine, London, WC1E 7HT UK

**Keywords:** Malaria, Vector control, Access, Coverage, Ownership, Sleeping units, Household indicators, Long-lasting insecticidal nets, Mass distribution, Côte d’Ivoire

## Abstract

**Background:**

To reduce malaria burden in Côte d'Ivoire, the Ministry of Health aims for 90% of its population to possess one long-lasting insecticidal net (LLIN) for every two persons by 2025. This study evaluated LLIN coverage two years after a mass distribution in central Côte d'Ivoire.

**Methods:**

A census was conducted in 43 villages. Data were collected on household geo-position, composition, number of sleeping units and LLINs owned. LLIN coverage was assessed using: 1/ownership; proportion of household with at least one LLIN; 2/household access; households with sufficient nets for every two persons and for every sleeping unit; and 3/population access; proportion of population with access to LLIN within households and sleeping units.

**Results:**

10,630 households (89.6% response rate) and 46,619 inhabitants were recruited. Household LLIN ownership was 63.8% (95% CI: 58.7–68.8). Household LLIN access was 37.6% (95% CI: 33.2–42.0) based on 1 LLIN per 2 persons and 37.1% (95% CI: 33.0–41.2) based on 1 net per sleeping unit. Population LLIN access based on 1 LLIN per 2 persons and 1 net per sleeping space was 53.3% (95% CI: 48.6–58.1) and 49.4% (95% CI: 45.1–53.6), respectively. Approximately 17% of households with access for every 2 persons did not have access by every sleeping unit and 9.7% of households with access by sleeping unit did not have access for every 2 persons. Households with adequate access by sleeping unit but not for every 2 persons tend to be larger with fewer sleeping units, and have children under 5 years old and female members. The largest households (>7 members) and households with at least one under-five member had the lowest access (20.8 and 27.3%, respectively).

**Conclusion:**

LLIN access was low in this area of intense indoor malaria transmission, 2 years after the last mass distribution campaign. Strategies are needed to improve LLINs coverage.

**Supplementary Information:**

The online version contains supplementary material available at 10.1186/s12936-025-05335-4.

## Background

Malaria is a life-threatening disease which causes unacceptably high levels of morbidity and mortality globally [1]. It is estimated that half of the global population remains at risk for malaria [2]. The entire population of Côte d’Ivoire is at-risk for malaria, with a prevalence of malaria infection estimated at 37% in children aged 6–59 months and an incidence of clinical malaria of 189.9 per 1000 in the general population [3–5].

Between 2000 and 2015, it is estimated that insecticidal vector control interventions, specifically the widespread scale-up of long-lasting insecticidal nets (LLINs) and indoor residual spraying, averted over three-quarters of clinical malaria cases [6]. LLINs are one of the most effective malaria prevention tools [7]. LLINs provide personal protection to the individual user (via a physical barrier reducing human-to-mosquito and mosquito-to-human transmission, and via the insecticide in the netting which can repel, kill and/or impact the fecundity of susceptible vectors) [2] and, at high levels of population coverage, community-level protection by reducing vector longevity and density [8–12].

Multiple studies have concluded that low LLIN access is the main driver of low LLIN usage [13–15]. Optimal coverage of LLINs can be achieved by providing sufficient access to LLINs alongside health promotion activities to maximize their use. The World Health Organization (WHO) recommends the distribution of one LLIN for every two persons for full coverage [16]. The National Malaria Control Programme (NMCP) of Côte d'Ivoire has the ambitious objective of having 90% of its population own one net for every two persons with at least 80% of those with LLINs using them by 2025 [4, 5]. The last mass distribution campaign took place in 2021 in Côte d'Ivoire.

The Roll Back Malaria Monitoring & Evaluation Reference Group (RBM MERG) has put forth household survey indicators for evaluating coverage and access to LLINs. Household LLIN ownership is defined as the proportion of households with at least one LLIN. Household LLIN access is the proportion of households with at least one LLIN for every two people. This indicator can be used to calculate the gap in intra-household LLIN ownership from the proportion of households with sufficient LLIN amongst those with a minimum of one LLIN. Finally, population LLIN access is the proportion of population with access to an LLIN within their household and it estimates the proportion of individuals who could potentially have slept under an LLIN, assuming one LLIN is used by two persons [17]. These RBM MERG indicators capture the fact that the mere presence of a LLIN in a household does not necessarily reflect individual access.

Some studies recommend the consideration of sleeping units, as an addition to the aforementioned RBM MERG indicators, to improve the accuracy of access estimates [18–20]. A study conducted in Tanzania found that RBM MERG indicators under-estimated LLIN access especially in lower socio-economic households which tend to be overcrowded [21], whereas another study from Ethiopia found that the RBM MERG indicators over-estimated LLIN coverage because it did not take into account units where people actually slept [22]. The Ethiopian study suggested the following access indicators for sleeping units: (1) household LLIN access defined as the proportion of households with at least one LLIN for every sleeping unit within the household; (2) intra-sleeping space ownership gap defined as the proportion of households with insufficient LLINs for every sleeping unit amongst those reporting at least 1 LLIN and (3) population access defined as the proportion of population with access to LLIN within their households based on one LLIN per sleeping unit. Discrepancies in LLIN access estimates between RBM MERG criteria and indicators adapted to sleeping units may occur. A household may have sufficient LLIN access by sleeping unit but not by number of sleeping units, indicating that more than 2 persons sleep per sleeping unit. Conversely, a household with sufficient access for every 2 people may not have sufficient access by sleeping units if 1 person sleeps in each sleeping unit.

Across several studies, some factors associated with higher LLIN ownership across some sub-Saharan African countries include an urban residence, having 2 or more sleeping units within a household, having an under-five or pregnant member in the household [23–25]. Lower LLIN ownership has been associated with increasing distance to nearest health centre, postulated to be linked to difficulties in acquiring LLINs as a consequence of remoteness and poorer road infrastructure [25–27]. Factors that impact mosquito density may affect the perception of household occupants about malaria risk and the decision to acquire or use LLINs. Mosquito density is highly associated with humidity [28–34], and certain landscape elements, including altitude, distance to nearest lake, and vegetation cover, measured by the normalized difference vegetation index (NDVI) [35–37]. However, there is large variability across studies in factors examined and the reported results, and there have been no studies in Côte d'Ivoire.

## Methods

### Study design and setting

**Fig. 1:**
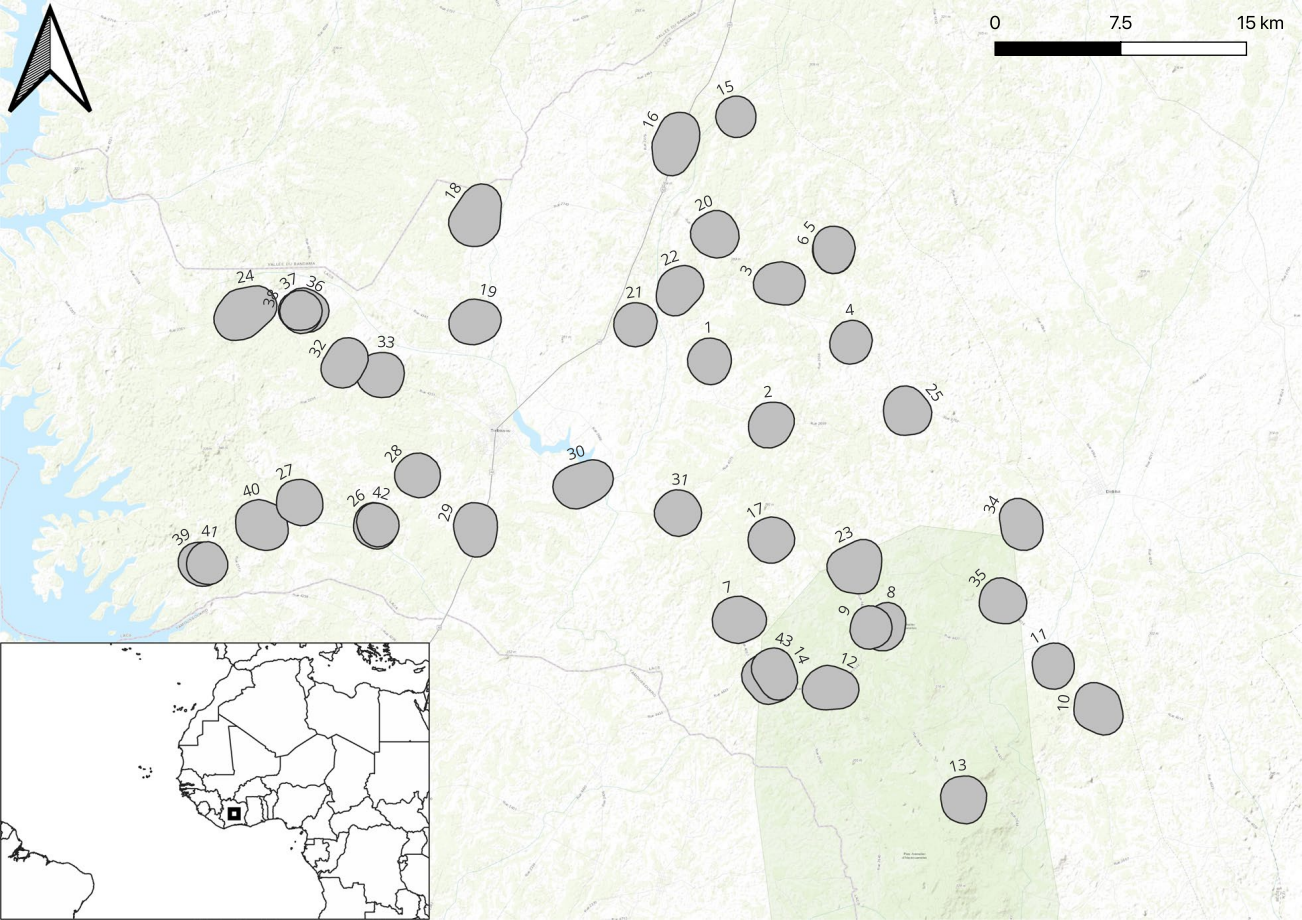
Map of study village boundaries, Tiebissou department, central Côte d’Ivoire, and inset map displaying the location of the study area in Africa. Map was created using QGIS 3.30.3 (Free Software Foundation Inc., Boston, MA, USA) using a basemap from ESRI (Redlands, CA, USA)

This paper reports the census data collected in preparation for a cluster randomized trial evaluating next-generation bed nets [38]. The census took place in 43 of the 110 villages in the department of Tiebissou (Gbêkê region, Lacs District), central Côte d’Ivoire (see Fig. 1) from June 8 to 27, 2023. These villages were selected based on the following criteria: having a minimum of 100 households with a minimum of 100 children under the age of 10 years (based on data from the health districts), within a 2-h drive from Tiebissou town, a minimum of 2 km between villages, and the acceptance of the hamlet leader and population to participate in the study. All households within selected villages were visited to have a count of the entire study population.

In 2021, the population of Tiebissou was estimated to be 116,321, spread over 2410 km^2^ [39]. The main vector control activity was the distribution of LLINs once every 3 years through mass distribution campaigns, with continuous distribution through antenatal and infant-welfare clinics. The last mass distribution campaign took place from March 11th to May 5th 2021, with an allocation of one LLIN for every 2 persons (no maximum cap per household). Deltamethrin-only nets were distributed in Tiebissou during this campaign. However, for the purpose of the current analysis, all net types were included in the assessment, irrespective of its brand. Nets were distributed from central points within villages. Household LLIN ownership was estimated at 76% in this district during a 2021 survey [3]. The prevalence of malaria infection in children under five in the Lac district based on microscopy and RDT were 32 and 51%, respectively in 2021 [3].

### Census process

A team of enumerators was trained in the consenting process, census procedures and electronic data capture. All housing structures (both inhabited and uninhabited) were visited in every village and geo-referenced using the Global Positioning System (GPS) function on a tablet. Written informed consent was obtained from an able adult. Data were collected using a questionnaire on the household member composition, number of LLINs, and number of sleeping units. Questionnaires were administered in French or Baoulé. Housing structures which looked inhabited but were vacant at the time of the visit were visited again 1 to 3 times to complete data collection. Data were cleaned and consistency checks were performed daily by a Data Manager.

### Operational definition of terms

A *sleeping unit* was defined in this study as an internal or external space (covered or uncovered) used temporarily or permanently for sleeping by one or more household members.

A *household* was a basic socio-economic unit in which the various members (related or unrelated) lived in the same house or compound, brought together their resources and jointly met their essential food and other vital needs.

A *factory-treated net with insecticides*, requiring no re-treatment and used for sleeping was considered as being a LLIN. LLINs are expected to last for 3–5 years or withstand 20 washes in the laboratory according to the World Health Organization [40].

The operational definitions used to determine access based on household indicators were from RBM MERG 2018 [17], whereas, the indicators adapted to sleeping units were adapted from an Ethiopian study, for easy comparability [22] and are summarized in Table 1.

**Table 1:** Coverage indicators and their calculation

Indicator	Definition	Calculation
Household LLIN ownership	Proportion of households with at least one LLIN	$$\frac{\text{Number of households reporting }\ge 1\text{ LLIN}}{Total number of households surveyed}$$
Household LLIN access (based on 1 LLIN per 2 persons)	Proportion of households with at least one LLIN for every 2 people	$$\frac{\text{Number of households with }\ge 1\text{ LLIN for every }2\text{ persons}}{Total number of households surveyed}$$
Intra-household ownership gap	Proportion of households with insufficient LLINs for every 2 persons amongst those with at least 1 LLIN	$1-\frac{\text{Number of households with }\ge 1\text{ LLIN for every }2\text{ persons}}{\text{Number of households reporting }\ge 1\text{ LLIN}}$
Population LLIN access (based on 1 LLIN per 2 persons)	Proportion of persons with access to a LLIN in their household, assuming each LLIN can be used by 2 persons	$$\frac{\text{Number of persons who could sleep under an LLIN if each LLIN is used by }2\text{ persons in the household }}{Total number of persons who spent the previous night in surveyed households}$$
Household LLIN access (based on 1 net per sleeping unit)	Proportion of households with sufficient LLINs to cover every sleeping unit within the household	$$\frac{\text{Number of households with enough nets for every sleeping unit}}{Total number of households surveyed}$$
Intra-sleeping unit ownership gap	Proportion of households with insufficient LLINs for every sleeping unit amongst those reporting at least 1 LLIN	$1-\frac{\text{Number of households with enough nets for every sleeping unit}}{\text{Number of households reporting }\ge 1\text{ LLIN}}$
Population access (based on 1 LLIN per sleeping unit)	Proportion of persons with access to a LLIN in their sleeping units, assuming each sleeping unit is covered by 1 LLIN	$$\frac{\text{Number of persons who could sleep under an LLIN if every sleeping unit was covered by }1\text{ LLIN }}{Total number of persons who spent the previous night in surveyed households}$$

### Data management and statistical analyses

Electronic data collection was performed using the Open Data Kit collect application installed on tablets. Data analyses was performed in STATA/SE version 18.0 (Stata Corp LP, College Station, TX). Households that did not consent either due to refusal or absence were not included in this analysis. Binary and categorical variables were summarized using proportions. Continuous variables were summarized using means (and standard deviations [SD]) or medians (and interquartile ranges [IQR]) as appropriate.

Variables were created for household LLIN ownership, household LLIN access (based on 1 LLIN per 2 persons and 1 LLIN per sleeping unit) and population access (based on 1 LLIN per 2 persons and 1 LLIN per sleeping unit). Details of all variables are shown in Table 2.

**Table 2:** Definition of variables

Variable	Description
Age (in years)	Data was categorized into three groups: <5, 5–15 and >15 years
Altitude (in meters)	Was categorized into 2 groups around the median: 148–209 (very low altitude) and 210–258 (low altitude)
Household LLIN access (based on 1 LLIN per 2 persons)	A new variable was generated which divided the total number of nets reported (regardless if used or not) in a household by total number of household inhabitants and re-categorized into a binary variable at a value of ≥0.5 (sufficient nets)
Household LLIN access (based on 1 net per sleeping unit)	A new variable was created by dividing the number of nets in a household by the total number of sleeping units in the household, and a binary variable created with households with a ratio of ≥1 tagged as having sufficient nets to cover each sleeping unit
Household LLIN ownership	The discrete variable, number of nets (whether used or not), was re-categorized as a binary variable (no net reported and ≥1 LLIN reported)
Household size	Was categorized into 3 equal groups with the following cut-offs: 1–3, 4–7 and 8–19 members
Nearest distance to a health facility (in km)	Was categorized into 3 equal groups with the following cut-offs: 0.4–3.6, 3.7–8.0 and 8.1–13.5
Nearest distance to a lake (in km)	Was categorized into 4 equal groups with the following cut-offs: < 4.2, 4.3–7.7, 7.8–11.6 and 11.7–36.0
Normalized difference vegetation index	Was categorized into 4 groups with the following cut-offs: low (71–5700), low-moderate (5701–7526), high-moderate (7527–8003) and high (8004- 9078) vegetation cover
Population access (based on 1 LLIN per 2 persons)	The number of potential net users was calculated for each household by multiplying the number of nets by 2 and dividing it by the household size (with a maximum value of 1) and the mean obtained across the study population
Population access (based on 1 LLIN per sleeping unit)	A new variable was generated which divided the total number of nets by the number of sleeping units in a household (with a maximum value of this ratio being 1) and then multiplied by household size to obtain how many persons would have been potentially covered within the sleeping spaces. The sum of these potential users was obtained and divided by the sum of household sizes
Population density (in persons/km^2^)	Was categorized into 2 groups around the median: 36–339 (very low) and 340–505 (low)

Each of the aforementioned indicators was calculated overall and for each village. The calculation of 95% confidence intervals (CI) for summary measures used svyset command to account for clustering by village.

Variables pertaining to household composition were generated by summing within each household the number of individuals with the characteristic of interest, re-categorizing the discrete values obtained into binary variables and obtaining relevant ratios when necessary. Household net ownership and access was assessed for different household compositions.

Geographic coordinates (datum: WSG84) of the households and health facilities were uploaded into QGIS v3.30.3. Earth Explorer database was accessed from United States Geological Survey to obtain Global Multi-resolution Terrain Elevation Data 2010 [41] and the EROS Visible Infrared Imaging Radiometer Suite [42] raster files, which represent altitude and NDVI, respectively. The WorldPop database was accessed to obtain population density data [43]. The ESRI database was accessed to obtain outline maps of lakes initially created by Messager et al*.* [44]. All coordinates were first projected to UTM30N. The ‘Distance to nearest hub’ tool was used to generate Euclidean distances in meters between each household and its nearest health facility and nearest lake, and the ‘Sample raster values’ was used to sample altitude, NDVI and population density at each household’s location.

## Results

**Fig. 2:**
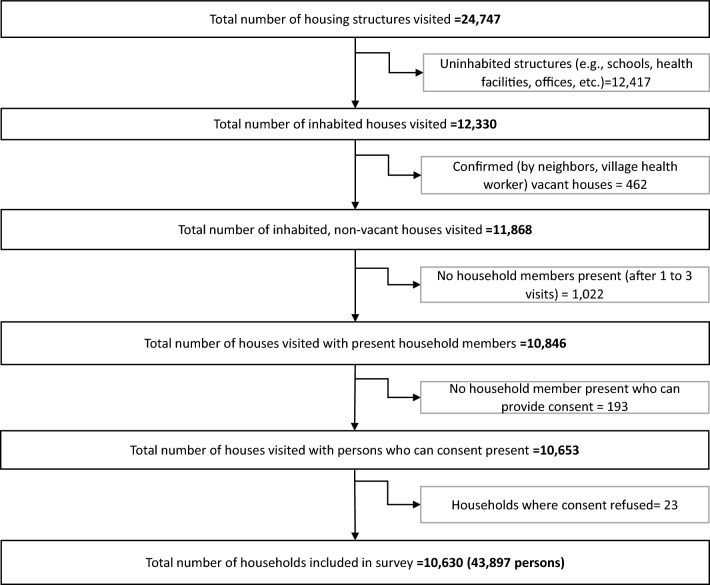
Flow chart of household inclusion

A total of 24,747 housing structures were identified with a total of 10,630 households (response rate of 89.6%) and 43,897 inhabitants. A flowchart of household inclusion for this analysis is shown in Fig. 2.

### Socio-demographic characteristics of the study population

Slightly over half of the study population were males (n = 22,335; 50.9%). Under-fives, school-aged children (aged 5 to 15) and adults (>15 years) made up 15.3, 29.4 and 55.3% of the study population, respectively. The median household size was 4 persons (interquartile range [IQR]: 2–6), with a median of 2 (IQR: 2–3) sleeping areas and 1 (IQR: 0–2) LLIN per household. The median distance from household to the closest health facility and the nearest lake were 3.65 km (IQR: 0.36–8.16) and 7.78 km (IQR: 4.06–11.34), respectively. The median altitude from sea level was 210 m (IQR: 191–235). The median population density was 129 people/km^2^ (IQR: 105–260). The socio-demographic and geo-spatial characteristics for each village are shown in Tables 3 and 4, respectively.

**Table 3:** Socio-demographic characteristics of study population

Village number	Number of households, n (%) N = 10,630	Median household size (IQR)	Number of males, n (%) N = 22,335	Number of females, n (%) N = 21,562	Under-fives, n (%) N = 6,697	School aged 5–15, n (%) N = 12,907	Adults > 15, n (%) N = 24,293	Median number of bed nets (IQR)	Median number of sleeping units (IQR)
1	281 (2.6)	4 (2–5)	520 (48.8)	546 (51.2)	171 (16.0)	292 (27.4)	603 (56.6)	1 (1–2)	2 (2–3)
2	230 (2.2)	4 (3–6)	441 (45.6)	526 (54.4)	189 (19.5)	263 (27.2)	515 (53.3)	1 (0–2)	2 (1–3)
3	370 (3.5)	4 (2–5)	712 (45.7)	845 (54.3)	255 (16.4)	422 (27.1)	880 (56.5)	1 (0–2)	2 (2–3)
4	137 (1.3)	3 (2–5)	248 (51.2)	236 (48.8)	82 (16.9)	141 (29.1)	261 (53.9)	1 (0–2)	2 (1–3)
5	178 (1.7)	4 (3–5)	326 (47.4)	362 (52.6)	110 (16.0)	198 (28.8)	380 (55.2)	1 (1–2)	2 (2–3)
6	123 (1.2)	4 (2–6)	254 (47.2)	284 (52.8)	62 (11.5)	165 (30.7)	311 (57.8)	1 (1–3)	3 (2–4)
7	369 (3.5)	4 (2–6)	808 (50.3)	798 (49.7)	239 (14.9)	434 (27.0)	933 (58.1)	1 (0–2)	2 (2–4)
8	135 (1.3)	4 (2–6)	267 (48.6)	283 (51.5)	84 (15.3)	180 (32.7)	286 (52.0)	1 (0–2)	2 (1–3)
9	81 (0.8)	4 (3–6)	196 (49.5)	200 (50.5)	57 (14.4)	143 (36.1)	196 (49.5)	1 (1–2)	3 (2–3)
10	291 (2.7)	4 (2–6)	598 (50.1)	596 (49.9)	204 (17.1)	353 (29.6)	637 (53.4)	1 (0–2)	2 (1–3)
11	179 (1.7)	5 (3–7)	457 (51.6)	429 (48.4)	167 (18.9)	267 (30.1)	452 (51.0)	2 (1–3)	3 (2–4)
12	172 (1.6)	4 (2–6)	323 (46.8)	367 (53.2)	109 (15.8)	176 (25.5)	405 (58.7)	2 (1–3)	2 (1–4)
13	194 (1.8)	4 (3–6)	406 (48.7)	428 (51.3)	145 (17.4)	208 (24.9)	481 (57.7)	0 (0–1)	2 (2–3)
14	194 (1.8)	4 (3–6)	426 (48.1)	459 (51.9)	119 (13.5)	276 (31.2)	490 (55.4)	0 (0–1)	3 (2–4)
15	109 (1.0)	4 (2–5)	211 (50.5)	207 (49.5)	73 (17.5)	135 (32.3)	210 (50.2)	1 (0–2)	2 (2–3)
16	157 (1.5)	4 (3–5)	337 (52.7)	303 (47.3)	66 (10.3)	232 (36.3)	342 (53.4)	1 (0–2)	2 (2–3)
17	277 (2.6)	3 (1–4)	449 (51.5)	423 (48.5)	131 (15.0)	270 (31.0)	471 (54.0)	0 (0–1)	2 (1–3)
18	354 (3.3)	5 (3–6)	890 (51.7)	830 (48.3)	290 (16.9)	485 (28.2)	945 (54.9)	1 (0–2)	3 (2–4)
19	320 (3.0)	4 (3–5)	704 (52.6)	634 (47.4)	214 (16.0)	399 (29.8)	725 (54.2)	1 (0–2)	2 (2–3)
20	340 (3.2)	4 (2–5)	713 (49.5)	727 (50.5)	249 (17.3)	394 (27.4)	797 (55.4)	1 (0–2)	2 (1–3)
21	284 (2.7)	4 (3–5)	619 (53.5)	539 (46.6)	176 (15.2)	287 (24.8)	695 (60.0)	0 (0–1)	2 (2–3)
22	167 (1.6)	4 (2–6)	365 (47.7)	400 (52.3)	123 (16.1)	202 (26.4)	440 (57.5)	2 (1–3)	3 (2–4)
23	396 (3.7)	4 (2–6)	791 (49.2)	816 (50.8)	247 (15.4)	479 (29.8)	881 (54.8)	0 (0–1)	2 (1–3)
24	474 (4.5)	3 (2–4)	867 (51.3)	824 (48.7)	240 (14.2)	529 (31.3)	922 (54.5)	2 (1–2)	2 (2–3)
25	372 (3.5)	4 (2–5)	705 (49.0)	733 (51.0)	263 (18.3)	431 (30.0)	744 (51.7)	0 (0–1)	2 (1–3)
26	194 (1.8)	4 (3–6)	477 (53.7)	412 (46.3)	93 (10.5)	287 (32.3)	509 (57.5)	2 (1–3)	3 (2–4)
27	238 (2.2)	5 (3–6)	638 (56.7)	488 (43.3)	150 (13.3)	344 (30.6)	632 (56.1)	1 (0–2)	3 (2–4)
28	157 (1.5)	3 (2–6)	289 (47.5)	320 (52.6)	101 (16.6)	170 (27.9)	338 (55.5)	0 (0–1)	2 (1–3)
29	339 (3.2)	4 (3–6)	854 (57.4)	633 (42.6)	186 (12.5)	481 (32.4)	820 (55.1)	1 (0–2)	2 (2–3)
30	351 (3.3)	4 (2–5)	678 (51.0)	652 (49.0)	250 (18.8)	317 (23.8)	763 (57.4)	1 (0–2)	2 (2–3)
31	461 (4.3)	4 (2–5)	971 (54.6)	809 (45.5)	247 (13.9)	535 (30.1)	998 (56.1)	1 (0–2)	2 (1–3)
32	287 (2.7)	4 (2–5)	536 (51.6)	503 (48.4)	91 (8.8)	272 (26.2)	676 (65.1)	1 (0–2)	2 (2–3)
33	379 (3.6)	4 (2–5)	684 (49.4)	700 (50.6)	140 (10.1)	365 (26.4)	879 (63.5)	1 (0–2)	2 (2–3)
34	203 (1.9)	3 (1–4)	302 (50.2)	300 (49.8)	105 (17.4)	174 (28.9)	323 (53.7)	0 (0–1)	2 (1–3)
35	349 (3.3)	3 (2–5)	652 (50.0)	652 (50.0)	237 (18.2)	413 (31.7)	654 (50.2)	1 (1–2)	2 (1–3)
36	38 (0.4)	3 (1–5)	75 (55.6)	60 (44.4)	23 (17.0)	50 (37.0)	62 (45.9)	1 (0–2)	2 (1–3)
37	129 (1.2)	4 (3–6)	323 (53.1)	285 (46.9)	99 (16.3)	197 (32.4)	312 (51.3)	0 (0–1)	3 (2–4)
38	83 (0.8)	3 (2–5)	156 (53.8)	134 (46.2)	38 (13.1)	120 (41.4)	132 (45.5)	0 (0–2)	2 (1–3)
39	276 (2.6)	5 (3–7)	750 (53.2)	659 (46.8)	229 (16.3)	425 (30.2)	755 (53.6)	1 (1–2)	3 (2–4)
40	497 (4.7)	5 (3–6)	1,304 (52.3)	1,190 (47.7)	382 (15.3)	769 (30.8)	1,343 (53.9)	1 (0–2)	3 (2–3)
41	70 (0.7)	5 (3–7)	184 (48.9)	192 (51.1)	61 (16.2)	134 (35.6)	181 (48.1)	1 (0–3)	3 (2–4)
42	97 (0.9)	5 (3–6)	245 (49.8)	247 (50.2)	82 (16.7)	151 (30.7)	259 (52.6)	1 (0–2)	3 (2–5)
43	298 (2.8)	3 (2–5)	584 (52.4)	531 (47.6)	118 (10.6)	342 (30.7)	655 (58.7)	1 (0–2)	2 (2–3)

**Table 4:** Study geo-spatial characteristics

Village number	Median distance from nearest health facility in km (IQR)	Median distance to the nearest lake in km (IQR)	Median altitude in metres (IQR)	Median population density in people/km^2^ (IQR)	Median normalized difference vegetation index (IQR)
1	4.5 (4.4–4.6)	10.3 (10.2–10.4)	222 (222–224)	222 (222–243)	7818 (7818–7818)
2	7.6 (7.5–7.7)	12.6 (12.6–12.7)	236 (231–237)	245 (245–245)	8592 (7325–8592)
3	0.3 (0.2–0.4)	7.6 (7.5–7.8)	250 (250–253)	405 (405–405)	5602 (5602–5602)
4	5.2 (5.1–5.3)	13.0 (12.9–13.1)	201 (201–204)	69 (69–69)	7426 (7426–7426)
5	3.5 (3.5–3.5)	9.6 (9.5–9.6)	252 (251–252)	97 (97–97)	7765 (7765–7765)
6	3.3 (3.2–3.4)	9.6 (9.5–9.6)	247 (247–251)	97 (97–97)	7765 (7765–7765)
7	0.2 (0.1–0.3)	4.1 (4.0–4.2)	186 (185–189)	333 (238–333)	8073 (8073–8073)
8	0.4 (0.3–0.4)	6.4 (6.4–6.5)	183 (182–184)	93 (93–93)	6788 (6788–6788)
9	0.8 (0.8–0.9)	5.8 (5.7–5.9)	178 (178–178)	105 (105–105)	7260 (7260–7260
10	8.2 (8.0–8.3)	12.5 (12.4–12.6)	180 (177–183)	118 (101–118)	6100 (6100–7886)
11	4.5 (4.5–4.6)	11.4 (11.3–11.5)	153 (153–157)	359 (359–359)	7766 (7766–7766)
12	4.0 (3.3–4.1)	3.4 (2.7–3.5)	192 (190–199)	46 (40–46)	6430 (6430–7571)
13	11.5 (11.3–11.6)	14.1 (14.0–14.1)	242 (229–242)	56 (56–56)	8170 (8170–8170)
14	0.2 (0.2–0.4)	0.5(0.5–0.6)	169 (162–177)	88 (88–88)	6944 (6944–8891)
15	10.3 (10.3–10.3)	6.2 (6.1–6.2)	246 (24–246)	119 (119–119)	574 (574–574)
16	8.8 (8.6–9.0)	3.8 (3.6–4.0)	243 (242–245)	120 (119–120)	6329 (6329–6329)
17	5.4 (5.3- 5.4)	7.9 (7.8–7.9)	207 (204–207)	167 (167–195)	8962 (5165–8962)
18	13.1 (13.0–13.2)	7.9 (7.8–8.2)	201 (194–203)	105 (105–105)	7928 (7867–7928)
19	12.4 (12.3–12.5)	5.0 (4.9–5.1)	187 (184–187)	417 (417–417)	7588 (7588–7588)
20	3.7 (3.7–3.8)	2.9 (2.8–2.9)	228 (228–228)	191 (191–191)	6464 (6464–7035)
21	3.5 (3.4–3.6)	8.7 (8.6–8.7)	234 (234–235)	160 (122–160)	184 (184–184)
22	0.2 (0.2–0.3)	4.9 (4.8–5.0)	222 (218–224)	171 (171–171)	5701 (5369–5701)
23	0.2 (0.1–0.2)	7.3 (7.2–7.4)	191 (190–194)	369 (369–369)	8004 (8004–8004)
24	12.4 (12.3–12.6)	26.4 (26.3–26.6)	246 (238–246)	111 (111–111)	7705 (7705–7705)
25	0.4 (0.2–0.5)	8.8 (8.7–9.0)	191 (191–196)	111 (111–111)	6487 (6487–6487)
26	0.4 (0.4–0.5)	7.5 (7.4–7.5)	223 (222–223)	96 (96–123)	7572 (7572–7572)
27	2.4 (2.3–2.5)	35.6 (35.5–35.7)	256 (252–256)	383 (383–383)	8442 (8442–8442)
28	3.7 (3.6–3.8)	3.5 (3.4–3.6)	231 (230–234)	96 (96–96)	111 (111–111)
29	0.1 (0.0–0.2)	6.9 (6.8–7.0)	210 (208–210)	467 (401–467)	4741 (4741–4741)
30	6.3 (6.3–6.4)	2.6 (2.5–2.7)	192 (190–193)	144 (144–144)	8176 (8176–8176)
31	0.3 (0.2–0.4)	8.1 (8.0–8.2)	209 (209–211)	302 (302–307)	5070 (5070–5070)
32	9.6 (9.4–9.7)	3.3 (3.2–3.5)	204 (201–204)	107 (107–107)	8426 (8426–8426)
33	8.6 (8.5–8.7)	2.9 (2.8–3.0)	214 (208–215)	129 (129–129)	8257 (8257–8257)
34	4.4 (4.3–4.6)	3.5 (3.3–3.6)	197 (190–199)	83 (67–83)	7454 (5893–7454)
35	0.3 (0.2–0.4)	8.0 (7.9–8.1)	163 (163–166)	236 (236–260)	7026 (4195–7026)
36	12.9 (12.8–12.9)	29.5 (29.4–29.6)	225 (225–226)	99 (99–99)	8252 (7233–8252)
37	12.9 (12.8–13.0)	5.0 (4.9–5.2)	225 (223–226)	71 (65–99)	6860 (6860–7233)
38	12.7 (12.6–12.8)	29.6 (29.5–29.7)	225 (223–225)	99 (99–99)	8252 (8252–8252)
39	4.5 (4.4–4.6)	34.2 (34.1–34.3)	240 (234–241)	505 (505–505)	8350 (8350–8350)
40	0.4 (0.3–0.5)	34.8 (34.7–35.1)	245 (244–245)	118 (118–122)	149 (149–151)
41	4.2 (4.1–4.2)	34.4 (34.4–34.5)	237 (230–237)	505 (505–505)	8350 (8350–8350)
42	0.5 (0.4–0.5)	7.2 (7.2–7.3)	222 (222–222	123 ( 96–123)	8487 (5349–8487)
43	0.4 (0.3–0.6)	0.8 (0.7–1.2)	165 (165–169)	88 (76–88)	6944 (6357–6944)

### LLIN coverage by household survey metrics

**Fig. 3:**
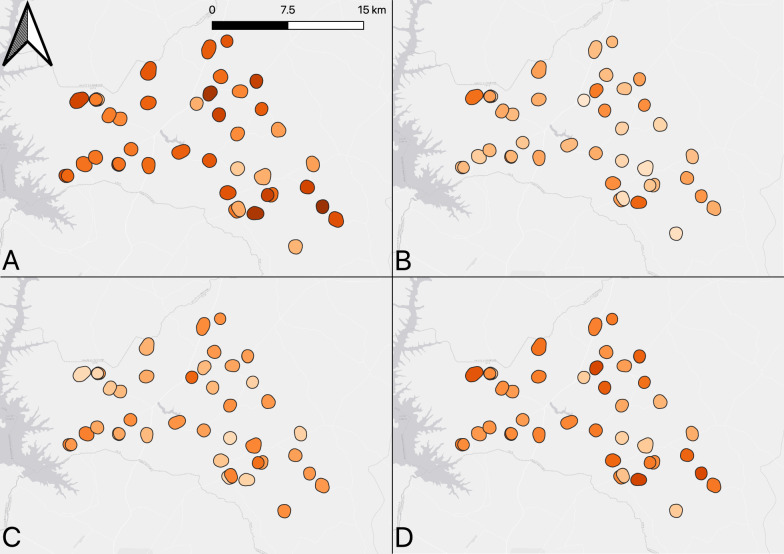
Map of LLIN coverage showing **A** Household LLIN ownership **B** Household LLIN access (based on one LLIN per 2 persons) **C** Intra-household ownership gap **D** Population LLIN access (based on one LLIN per 2 persons) within the 42 study villages. Map was created using QGIS 3.30.3 (Free Software Foundation Inc., Boston, MA, USA) using a basemap from ESRI (Redlands, CA, USA). Legend:

The results of LLIN coverage using household survey metrics for each village is shown in Fig. 3 and Supplementary Table 1. Overall, household LLIN ownership was 63.8% (95% CI: 58.7–68.8), with a range of 28.5–95.9% by village. Household LLIN access was 37.6% (95% CI: 33.2–42.0) with values varying from 18.0 to 65.0% by village. The intra-household ownership gap was 41.0% (95% CI: 37.1–45.0), with a range of 20.3–66.1%. Population LLIN access (based on 1 LLIN per 2 persons) was 53.3% (95% CI: 48.6–58.1), with a range from 26.4 to 83.1% by village.

### LLIN coverage by sleeping units

**Fig. 4:**
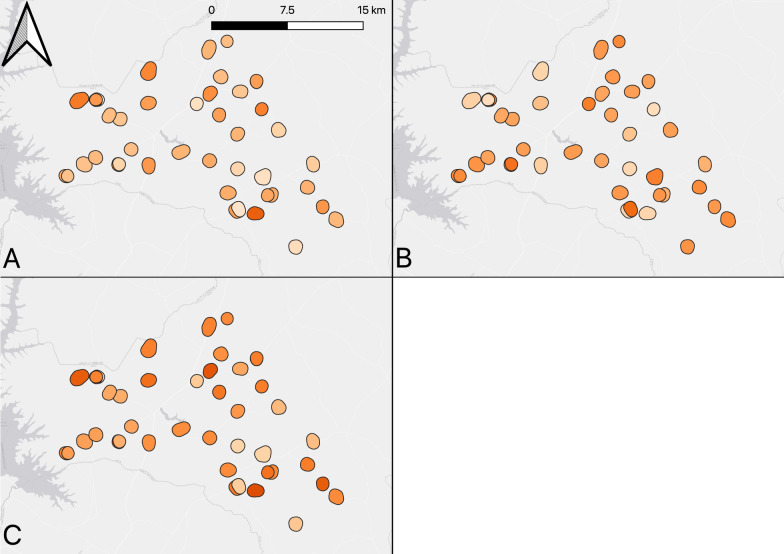
Map of LLIN coverage showing **A** Household LLIN access (based on one LLIN per sleeping unit) **B** Intra-sleeping unit ownership gap **C** Population LLIN access (based on one LLIN per sleeping unit) within the 42 study villages. Map was created using QGIS 3.30.3 (Free Software Foundation Inc., Boston, MA, USA) using a basemap from ESRI (Redlands, CA, USA). Legend:

The results of LLIN coverage by metrics adapted to sleeping units for each village is shown in Fig. 4 and in Supplementary Table 1. Household LLIN access was 37.1% (95% CI: 33.0–41.2), with a range of 14.0–68.6%. The intra-sleeping unit ownership gap was 41.8% (95% CI: 38.2–45.5), with a range of 18.2–64.1% by village. Population LLIN access (based on 1 LLIN per sleeping unit) was 49.4% (95% CI: 45.1–53.6), with a range from 24.6–75.9% by village.

### Household LLIN access based on one LLIN for every 2 persons compared to one LLIN per sleeping unit

**Fig. 5:**
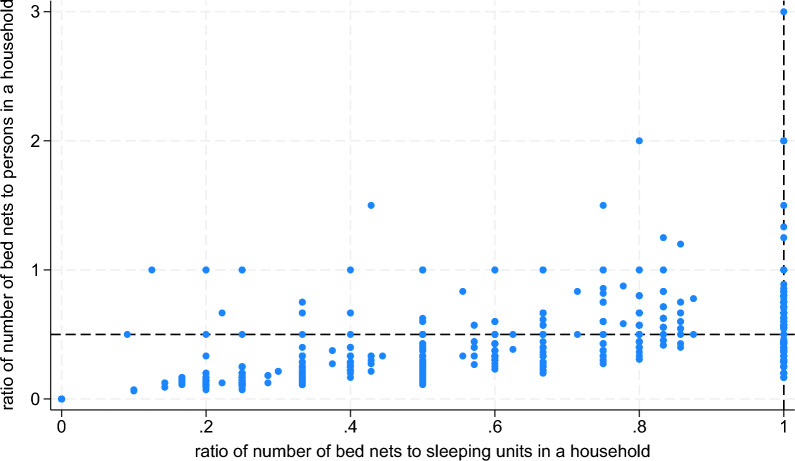
Scatterplot of the ratio of the number of LLINs to the household size to the ratio of the number of LLINs to the number of sleeping units for each household. Footnote: The y-axis reference line is the line of sufficiency of LLINs for every 2 persons at a ratio of 0.5 whereas the x-axis reference line is at 1 for sufficient LLINs for every sleeping unit

Figure 5 shows that most households (n = 5991, 56.4%) had neither sufficient nets per 2 persons nor for every sleeping unit within the household. A total of 3300 households (31.0%) had sufficient nets per 2 persons and for every sleeping unit. Furthermore, 17.4% (n = 696) of households with sufficient nets for every 2 persons did not have sufficient nets for every sleeping unit and 9.7% (n = 643) of households with sufficient nets for every sleeping unit did not have sufficient nets for every 2 persons. As shown in Table 5, households that have sufficient LLIN access by sleeping unit but not for every 2 persons tend to be larger and have fewer sleeping units, while households with LLIN access per 2 persons but not by sleeping unit tend to have more households with no children under the age of five and no females.

**Table 5:** Characteristics of households with household LLIN access discordance by sleeping unit and for every 2 persons

	Adequate access per 2 persons but not by sleeping unit N = 696	Adequate access by sleeping unit but not per 2 persons N = 643
Mean household size (standard deviation)	4.4 (2.2)	5.1 (2.07)
Mean number of sleeping units	3.8 (1.7)	1.9 (0.9)
Households with no female member	11.2%	1.7%
Households with no under-five member	66.0%	22.6%

### Household composition and LLIN coverage

Table 6 shows that while LLIN ownership was similar for different household compositions, there was more variation in LLIN access. Larger households (≥8 members) and households with at least one member aged under 5 years had the lowest household LLIN access at 20.8 and 27.3%, respectively.

**Table 6:** Household composition, geo-spatial metrics and LLIN coverage

	Categories	Number of households with at least one LLIN present in household, n (%)	Number of households with one LLIN for every 2 persons in household, n (%)
Households with at least one member aged under five	Yes	3069 (67.2)	1246 (27.3)
	No	3709 (61.2)	2750 (45.4)
Households with at least one school-aged member (5–15 years)	Yes	3996 (65.3)	2243 (34.6)
	No	2782 (61.6)	2574 (51.8)
Households with adults only	Yes	8 (61.5)	8 (57.1)
	No	6770 (63.8)	4809 (42.1)
Ratio of children to adults in a household	<1	3446 (61.8)	2382 (42.7)
	≥1	3324 (65.9)	1607 (31.9)
Households with at least one male member	Yes	6050 (64.1)	4104 (40.4)
	No	728 (61.4)	713 (55.2)
Households with at least one female member	Yes	5904 (64.7)	3197 (35.0)
	No	874 (58.0)	799 (53.0)
Ratio of females to males in a household	<1	2884 (63.2)	1630 (35.7)
	≥1	3166 (64.8)	1759 (36.0)
Household size	1–3	2913 (61.6)	2294 (48.5)
	4–7	3284 (65.1)	1526 (30.2)
	8–19	580 (68.4)	176 (20.8)
Closest distance between household and health facility (in km)	<0.4	1647 (61.9)	943 (35.5)
	0.4–3.6	1753 (65.1)	1003 (37.3)
	3.7–8.0	1647 (64.5)	936 (36.6)
	8.1–13.5	1724 (63.7)	1107 (40.9)
Nearest distance to a lake (in km)	<4.2	1748 (61.5)	1097 (38.6)
	4.3–7.7	1575 (63.3)	929 (37.4)
	7.8–11.6	1778 (64.8)	1000 (36.4)
	11.7–36.0	1670 (65.8)	963 (37.9)
Altitude (in metres) (<500 m is low altitude)	<210	3248 (62.7)	1969 (38.0)
	≥210	3523 (64.8)	2020 (37.2)
Population density (in persons/km^2^)	<340 (very low)	5558 (64.3)	3328 (38.5)
	≥340 (low)	1213 (61.6)	661 (33.6)
Normalized difference vegetation index	71–5700 (low)	1593 (62.1)	853 (33.4)
	5701–7526 (low-moderate)	1725 (62.6)	1024 (37.2)
	7527–8003 (high-moderate)	1786 (76.9)	1128 (48.6)
	8004–9078 (high)	1667 (56.2)	979 (33.0)

### Geo-spatial metrics and LLIN coverage

LLIN coverage did not vary either by shortest distance intervals from households to health facilities and lakes, altitude or population density. Household LLIN ownership and access was higher at a high-moderate range of vegetation cover (Table 6). Also, variability in LLIN coverage indicators by village was not explained by village size, shortest distance intervals from households to health facilities and lakes, altitude, population density or vegetation cover.

## Discussion

This study reports coverage of LLINs in an area of central Côte d’Ivoire with intense indoor malaria transmission using RBM MERG indicators and indicators adapted to sleeping units. Two years post-distribution, household LLIN ownership was moderate at 63.8%, while household LLIN access (based on 1 net per 2 persons) was low at 37.6, and 53.3% of the population had access to LLINs. Household access based on sleeping units was similar (37.1%), while population access was slightly lower (49.4%). Close to one-tenth (9.7%) of households with access by sleeping unit did not have access for every 2 persons and two-fifths (17.4%) of households with access for every 2 persons did not have access by sleeping unit. Households with access by sleeping unit but not for every 2 persons tend to be larger with fewer sleeping units, and have more female and under-five members. Access was lower as the number of household members increased and in households that had younger children. LLIN coverage did not vary either by shortest distance intervals from households to health facilities and lakes, altitude or population density, but access was higher at a high-moderate range of vegetation cover.

In this study, household LLIN ownership (64%) was lower than during the 2021 Demographic Health Survey (DHS), which reported 76% household LLIN ownership in the Lac District [3]. This difference was not unexpected as the DHS survey was done 6 months following the mass LLIN distribution campaign, while the present study was done 24 months after. Other studies done between 1 and 4 years post mass distribution campaigns had various household LLIN ownership; Tanzania (74.5%) [45], Nigeria (56%) (46), Burkina Faso (33%) [47], Guinea (44%) [48], Benin (95.8%) [49], Ethiopia (92.6%) [22] and Cameroon (73%) [50]. These differences could be explained by differences in proximity of coverage surveys to mass distributions, the frequencies of and strategies used for mass net distribution across countries (central point versus door-to-door distribution), the use of school-based top-up campaigns (not implemented in Cote d’Ivoire), as well as differences in LLIN durability.

Household and population LLIN access based on 1 net per 2 persons was 37.6 and 59.3% respectively. Household and population access in other countries 1 to 4 years after a mass distribution campaign were within range of those found in our study; Nigeria (25 and 43%, respectively) [46], Benin (55.9 and 79.5%, respectively) [49], Ethiopia (50.3 and 78.6%, respectively) [22], Cameroon (41 and 59%, respectively)[50] and Tanzania (41% household LLIN access) [51]. During the 2021 DHS survey in Côte d’Ivoire, household LLIN access was 51% [3]. This reduction in access 2 years after the LLIN distribution campaign could be attributed to issues with LLIN durability. According to the WHO, LLINs are expected to be in serviceable conditions within households for at least 3 years [40]. A study in Tanzania found that after permethrin-only LLINs had been used for 20 months in households, 100% of nets had at least 1 hole and only 63% of LLINs were in serviceable conditions [52]. Another study from Benin found that the number of serviceable deltamethrin-only nets dropped to 29 to 33% of nets after 3 years of field use [53].

In this study, household LLIN ownership was moderate and did not vary much by household composition, meanwhile access was considerably lower. This could suggest that household access may be a better indicator for LLIN coverage than LLIN ownership because LLIN ownership does not seem to change much with time after a mass distribution campaign. Also, having one LLIN does not necessarily translate into every household member having access to an LLIN. Household access (based on 1 net per 2 persons) was particularly low in households with at least one under-five member and in larger households. It was observed that larger households tend to have children and females (of any age). Adult women and children might be easier to reach with at least 1 net through continuous distribution channels. Also, LLINs may degrade faster in larger households, especially where there are children, as net care may not be as diligent and children might be more likely to sleep on mats rather than beds, which could result in greater damage to the nets., Larger households also tend to have fewer sleeping units and hence may be more likely covered with fewer nets within the sleeping units due to overcrowding, though not covered sufficiently for every 2 persons.

Even though in this study wide variability in LLIN coverage indicators by village was not explained by village size, shortest distance intervals from households to health facilities and lakes, altitude, population density or vegetation cover, investigating the reasons for this could inform future mass distribution strategies.

This is the first study of its type to describe LLIN coverage in this region of Côte d’Ivoire. An exhaustive sample of all the households was used and the response rate was very high, reducing the probability of selection bias due to non-response. A weakness of this study was the attachment of the census to an imminent net distribution activity which may have caused respondents to over-report number of household inhabitants and/or under-report the number of nets owned.

## Conclusions

Two years after a mass distribution, LLIN access was low in this rural region of Côte d’Ivoire, with considerable variability by village. To achieve and maintain the goal of having 90% of Côte d’Ivoire’s population with sufficient LLINs for every 2 individuals by 2025, continuous LLIN distribution through various channels, including schools, should be implemented to uphold coverage of mass distribution campaigns. Moreover, further net distribution and communication strategies should be implemented to reach specific groups such as adult-only households who are not typically targeted by routine LLIN distribution at health facilities. Finally, the NMCP should consider using a combination of indicators to assess LLIN coverage to have a more complete picture of the gaps in LLIN coverage which is the greatest barrier to use. A combination of these recommendations could significantly improve LLIN access and consequently usage, thereby contributing to the reduction of malaria morbidity and mortality in Côte d’Ivoire.

## Supplementary Information

Additional file 1.

## Data Availability

No datasets were generated or analysed during the current study.

## Abbreviations

CI: Confidence interval
DHS: Demographic Health Survey
GPS: Global Positioning System
LLIN: Long-lasting insecticidal nets
NDVI: Normalized Difference Vegetation Index
NMCP: National Malaria Control Programme
RBM MERG: Roll Back Malaria Monitoring & Evaluation Reference Group
RDT: Rapid diagnostic test
