# Supplementary material for: Evaluation of household coverage with long-lasting insecticidal nets in central Côte d’Ivoire
Source: Malar J. 2025 Mar 29;24:104. doi: 10.1186/s12936-025-05335-4 (PMC11955107; doi:10.1186/s12936-025-05335-4)
Supplement: Supplementary file 1 — Additional file 1. [file 12936_2025_5335_MOESM1_ESM.docx]

**Supplementary Table 1: Long-lasting insecticidal net coverage**

| **Village number** | **Household indicators** | | | | **Sleeping unit indicators** | | |
| --- | --- | --- | --- | --- | --- | --- | --- |
|  | **Households with ≥1 net, n(%)**  **N=10,630** | **Households with 1 net for every 2 persons, n(%)**  **N=10,630** | **Intra-household ownership gap, n(%)**  **N= 6,778** | **Population with access to a net in their household, n(%)**  **N=43,900** | **Households with LLIN for every sleeping unit within the household, n(%)**  **N=10,630** | **Intra-sleeping unit ownership gap, n(%)**  **N=6,778** | **Population with access to a LLIN in their sleeping units in the household, n(%)**  **N=43,900** |
| 1 | 225 (80.07) | 146 (51.96) | 79 (35.11) | 806 (75.61) | 126 (44.84) | 99 (44.00) | 635 (59.54) |
| 2 | 120 (52.17) | 59 (25.65) | 61 (50.83) | 480 (49.64) | 83 (36.09) | 37 (30.83) | 466 (48.20) |
| 3 | 202 (54.59) | 116 (31.35) | 86 (42.57) | 792 (50.87) | 111 (30.00) | 91 (45.05) | 645 (41.41) |
| 4 | 95 (69.34) | 71 (51.82) | 24 (25.26) | 348 (72.72) | 77 (56.20) | 18 (18.95) | 279 (58.00) |
| 5 | 146 (82.02) | 80 (44.94) | 66 (45.21) | 490 (71.22) | 81 (45.51) | 65 (44.52) | 398 (57.89) |
| 6 | 106 (86.18) | 61 (49.59) | 45 (42.45) | 442 (82.16) | 52 (42.28) | 54 (50.94) | 326 (60.65) |
| 7 | 272 (73.71) | 189 (51.22) | 83 (30.51) | 1104 (68.74) | 144 (39.02) | 128 (47.06) | 847 (52.72) |
| 8 | 87 (64.44) | 47 (34.81) | 40 (45.98) | 318 (57.82) | 54 (40.00) | 33 (37.93) | 288 (52.36) |
| 9 | 66 (81.48) | 25 (30.86) | 41 (62.12) | 240 (60.60) | 34 (41.98) | 32 (48.48) | 242 (61.02) |
| 10 | 216 (74.23) | 114 (39.18) | 102 (47.22) | 788 (66.00) | 103 (35.40) | 113 (52.31) | 623 (52.20) |
| 11 | 165 (92.18) | 87 (48.60) | 78 (47.27) | 746 (84.20) | 86 (48.04) | 79 (47.88) | 619 (69.83) |
| 12 | 153 (88.95) | 115 (66.86) | 38 (24.84) | 662 (95.94) | 118 (68.60) | 35 (22.88) | 518 (75.09) |
| 13 | 70 (36.08) | 34 (17.53) | 36 (51.43) | 294 (35.25) | 35 (18.04) | 35 (50.00) | 250 (30.00) |
| 14 | 78 (40.21) | 35 (18.04) | 43 (55.13) | 312 (35.25) | 28 (14.43) | 50 (64.10) | 244 (27.55) |
| 15 | 79 (72.48) | 39 (35.78) | 40 (50.63) | 274 (65.43) | 37 (33.94) | 42 (53.16) | 225 (53.63) |
| 16 | 109 (69.43) | 52 (33.12) | 57 (52.29) | 414 (64.69) | 57 (36.31) | 52 (47.71) | 354 (55.30) |
| 17 | 79 (28.52) | 63 (22.74) | 16 (20.25) | 278 (31.88) | 62 (22.38) | 17 (21.52) | 203 (23.29) |
| 18 | 253 (71.47) | 158 (44.63) | 95 (37.55) | 1038 (60.35) | 193 (54.52) | 60 (23.72) | 966 (56.19) |
| 19 | 215 (67.19) | 127 (39.69) | 88 (40.93) | 956 (71.31) | 146 (45.62) | 69 (32.09) | 838 (62.50) |
| 20 | 217 (63.82) | 114 (33.53) | 103 (47.47) | 832 (57.78) | 114 (33.53) | 103 (47.47) | 723 (50.21) |
| 21 | 109 (38.38) | 37 (13.03) | 72 (66.06) | 360 (31.09) | 46 (16.20) | 63 (57.80) | 326 (28.17) |
| 22 | 149 (89.22) | 98 (58.68) | 51 (34.23) | 726 (94.90) | 86 (51.50) | 63 (42.28) | 557 (72.77) |
| 23 | 154 (38.89) | 70 (17.68) | 84 (54.55) | 468 (29.09) | 67 (16.92) | 87 (56.49) | 396 (24.59) |
| 24 | 375 (79.11) | 297 (62.66) | 78 (20.80) | 1504 (89.41) | 278 (58.65) | 97 (25.87) | 1169 (69.30) |
| 25 | 169 (45.43) | 89 (23.92) | 80 (47.34) | 588 (40.99) | 93 (25.00) | 76 (44.97) | 498 (34.73) |
| 26 | 186 (95.88) | 126 (64.95) | 60 (32.26) | 860 (96.74) | 107 (55.15) | 79 (42.47) | 674 (75.85) |
| 27 | 143 (60.08) | 84 (35.29) | 59 (41.26) | 640 (56.84) | 81 (34.03) | 62 (43.36) | 506 (44.96) |
| 28 | 94 (59.87) | 46 (29.30) | 48 (51.06) | 298 (48.93) | 51 (32.48) | 43 (45.74) | 258 (42.40) |
| 29 | 219 (64.60) | 143 (42.18) | 76 (34.70) | 834 (56.09) | 160 (47.20) | 59 (26.94) | 755 (50.80) |
| 30 | 239 (68.09) | 120 (34.19) | 119 (49.79) | 784 (58.95) | 127 (36.18) | 112 (46.86) | 680 (51.16) |
| 31 | 329 (71.37) | 183 (39.70) | 146 (44.38) | 1096 (61.57) | 189 (41.00) | 140 (42.55) | 936 (52.61) |
| 32 | 164 (57.14) | 115 (40.07) | 49 (29.88) | 550 (52.94) | 96 (33.45) | 68 (41.46) | 428 (41.20) |
| 33 | 201 (53.03) | 132 (34.83) | 69 (34.33) | 702 (50.70) | 114 (30.08) | 87 (43.28) | 550 (39.70) |
| 34 | 90 (44.33) | 67 (33.00) | 23 (25.56) | 282 (46.84) | 56 (27.59) | 34 (37.78) | 199 (33.03) |
| 35 | 278 (79.66) | 155 (44.41) | 123 (44.24) | 906 (69.48) | 132 (37.82) | 146 (52.52) | 756 (57.97) |
| 36 | 22 (57.89) | 17 (44.74) | 5 (22.73) | 88 (65.19) | 18 (47.37) | 4 (18.18) | 75 (55.31) |
| 37 | 46 (35.66) | 23 (17.83) | 23 (50.00) | 204 (33.50) | 18 (13.95) | 28 (60.87) | 150 (24.66) |
| 38 | 57 (68.67) | 36 (43.37) | 21 (36.84) | 178 (61.03) | 40 (48.19) | 17 (29.82) | 158 (54.01) |
| 39 | 211 (76.45) | 94 (34.06) | 117 (55.45) | 870 (61.75) | 105 (38.04) | 106 (50.24) | 779 (55.28) |
| 40 | 308 (61.97) | 135 (27.16) | 173 (56.17) | 1214 (48.67) | 156 (31.39) | 152 (49.35) | 1114 (44.67) |
| 41 | 46 (65.71) | 24 (34.29) | 22 (47.83) | 212 (56.38) | 22 (31.43) | 24 (52.17) | 172 (45.78) |
| 42 | 60 (61.86) | 35 (36.08) | 25 (41.67) | 250 (50.81) | 23 (23.71) | 37 (61.67) | 192 (38.99) |
| 43 | 176 (59.06) | 138 (46.31) | 38 (21.59) | 808 (72.37) | 137 (45.97) | 39 (22.16) | 648 (58.03) |
